# G Protein-Coupled Receptors in Cell Signaling Transduction

**DOI:** 10.3390/ijms25010291

**Published:** 2023-12-25

**Authors:** Sandra Beer-Hammer, Ines Liebscher

**Affiliations:** 1 Department of Pharmacology, Experimental Therapy and Toxicology, Institute of Experimental and Clinical Pharmacology and Pharmacogenomic and ICePhA, University of Tübingen, D-72074 Tübingen, Germany; 2 Rudolf Schönheimer Institute of Biochemistry, University of Leipzig, 04103 Leipzig, Germany

G protein-coupled receptors (GPCRs) and their downstream signaling pathways are critical targets for current pharmacotherapy. More than one third of the drugs in use today act on GPCRs either as agonists, inverse agonists, or antagonists, although the GPCR receptor superfamily accounts for only 12% of drug targets [1,2,3]. More drugs interfere with GPCRs than with any other protein or cellular structure, including protein kinases or ion channels. Thus, GPCRs currently serve as extraordinary receptors in drug therapy. However, their therapeutic potential is not exhaustive, especially since recent findings point to new pharmacotherapeutically relevant regulatory mechanisms on the activator as well as in the signaling level. Allosteric modulators can influence the activation states of a GPCR and, particularly, hitherto largely unknown non-classical or also termed non-canonical regulatory mechanisms of GPCR signaling have been identified that can either modulate canonical signaling by G proteins or trigger unrelated signaling events [4,5,6,7,8]. These pathways are governed by other receptor classes than GPCRs, and various non-receptor regulators, including activators of G protein signaling (AGS) proteins or phosphate transferring nucleoside diphosphate kinases (NDPKs). In addition, GPCR adapters such as arrestins and regulators of G protein signaling (RGS) can exert additional functions distinct from the inactivation of G protein signaling.

The intention of this Special Issue was to highlight the (patho)physiological relevance of canonical and non-canonical GPCR signaling in biological processes, such as muscle contractility, neuronal procession, immune cell function, migration, or aging, which are crucial for major human diseases, including metabolic disorders, cancer, cardiovascular diseases, immune responses, or (neuro-)sensory defects. Further, we collected manuscripts that described the identification of new GPCR–adapter molecule interaction modes and assemblies, studied receptor dimerization, and evaluated the effects of known ligands and allosteric modulators.
